# Regorafenib vs trifluridine/tipiracil for metastatic colorectal cancer refractory to standard chemotherapies: A multicenter retrospective comparison study in Japan

**DOI:** 10.1371/journal.pone.0234314

**Published:** 2020-06-12

**Authors:** Misato Ogata, Masahito Kotaka, Takatsugu Ogata, Yukimasa Hatachi, Hisateru Yasui, Takeshi Kato, Akihito Tsuji, Hironaga Satake

**Affiliations:** 1 Department of Medical Oncology, Kobe City Medical Center General Hospital, Kobe City, Hyogo, Japan; 2 Gastrointestinal Cancer Center, Sano Hospital, Kobe City, Hyogo, Japan; 3 Department of Surgery, Osaka National Hospital, Osaka City, Osaka, Japan; 4 Department of Clinical Oncology, Faculty of Medicine, Kagawa University, Kitagun, Kagawa, Japan; 5 Cancer Treatment Center, Kansai Medical University Hospital, Hirakata City, Osaka, Japan; Chang Gung Memorial Hospital at Linkou, TAIWAN

## Abstract

Regorafenib (REG) and trifluridine/tipiracil (FTD/TPI) showed survival benefits in metastatic colorectal cancer patients previously treated with standard chemotherapies; therefore, we compared the efficacy and safety of these two treatments. Patients with metastatic colorectal cancer treated with REG or FTD/TPI as a salvage-line therapy from May 2014 to December 2017 were included. We retrospectively analyzed long-term survival, safety, and clinical outcomes. Among 134 patients, 57 and 77 received REG and FTD/TPI, respectively. The REG group received more prior systemic chemotherapies and significantly more frequent additional chemotherapies than the FTD/TPI group did. The median follow-up was 6.2 months, whereas the median overall survival was 9.9 and 11.4 months in the REG and FTD/TPI groups, respectively (hazard ratio = 0.954, p = 0.837). The median progression-free survival was 2.0 and 3.3 months in the REG and FTD/TPI groups, respectively (hazard ratio = 0.52, p = 0.00047), indicating significant differences, whereas the objective response and disease control rates did not differ. The median overall survival of patients with additional subsequent chemotherapies after disease progression was longer than that of patients without additional chemotherapy. The most frequent grade ≥3 adverse events were hypertension and neutropenia in the REG and FTD/TPI groups, respectively. Our study suggested that sequential use of both drugs may prolong survival.

## Introduction

Remarkable progress in the development of efficacious drugs for metastatic colorectal cancer (mCRC) has prolonged the median overall survival (OS) from first-line therapy up to 30 months [1]. However, mCRC is still one of the major causes of cancer-related death, and many patients experience a phase that is refractory to conventional cytotoxic and molecularly targeted agents [2]. After failure of the first few lines of combination chemotherapy including treatment with fluoropyrimidine, oxaliplatin, irinotecan, anti-vascular endothelial growth factor, and anti-epidermal growth factor receptor (EGFR) antibody, standard treatment options are limited. To date, regorafenib (REG) and trifluridine/tipiracil (FTD/TPI) are the only drugs that have been approved as late-line treatment drugs for patients with mCRC based on a global randomized phase III trial [3,4]. REG is a multikinase inhibitor that is active against several angiogenic receptor tyrosine kinases (RTKs), oncogenic RTKs, stromal RTKs, and intracellular signaling kinases [4]. FTD/TPI is a combination of trifluridine and tipiracil hydrochloride. Trifluridine is a thymidine-based nucleic acid analog, and tipiracil hydrochloride is a thymidine phosphorylase inhibitor, which enables the maintenance of high trifluridine concentration. Since REG and FTD/TPI have not been directly compared in a clinical trial despite their similarity, the appropriate selection of REG or FTD/TPI for mCRC treatment remains under discussion. The aim of this study was to compare the real-world efficacy and safety of FTD/TPI and REG in patients with mCRC refractory to standard chemotherapies, and to suggest predictive or prognostic factors for treatment with these two drugs.

## Materials and methods

### Patient selection

We retrospectively collected the clinical data of 134 patients with mCRC who were treated with REG or FTD/TPI as salvage-line therapy at two Japanese tertiary referral centers, Kobe City Medical Center General Hospital and Sano Hospital, from May 2014 to December 2017. This retrospective analysis was approved by the Ethics Committee of each institution, and the requirement to obtain informed consent was waived because this was a retrospective investigation. Long-term survival, safety, and clinical outcomes were evaluated. Major inclusion criteria of this analysis were as follows: histologically confirmed colorectal adenocarcinoma; refractoriness to fluoropyrimidine, oxaliplatin, irinotecan, bevacizumab, and anti-EGFR antibody (limited to wild-type *KRAS* or *RAS*); measurable or evaluable lesion; age ≥20 years old; ECOG PS 0 to 2; adequate organ function. Some of the patients in the FTD/TPI group used a combination regimen with bevacizumab or panitumumab.

### Treatments and assessments

FTD/TPI was administered at 35 mg/m^2^ orally, twice daily, on days 1–5 and 8–12 in each month. REG was administered at 160 mg once daily on days 1–21 in each month, but in some cases was started at a lower dose, considering the patient’s condition (17 cases; 32% of all the REG group patients, started at 120 mg/day and 1 case started at 80 mg/day.). Treatment was discontinued if the tumor progressed, severe toxicity occurred, or under the instructions of the treating physician for any reason.

Patients’ characteristics, adverse events, treatment response, progression-free survival (PFS), and OS were analyzed using data collected from medical records. The response evaluation criteria for this study were based on response evaluation criteria in solid tumor (RECIST) version 1.1, and adverse events were defined based on the National Cancer Institute—Common Toxicity Criteria (NCI-CTC) version 4.0. The objective response rates (ORR) included complete response (CR) plus partial response (PR), and disease control rate (DCR) included CR plus PR plus stable disease (SD). OS was defined as the time from the first administration of chemotherapy to death from any cause or last confirmation of survival. PFS was defined as the time from the first administration of chemotherapy to the first documentation of disease progression, subsequent therapy, or death from any cause.

### Statistical analysis

Analysis was performed using R. Survival analysis was performed using the Kaplan-Meier method. The Pearson chi-squared test or Fisher’s exact test was used to analyze the categorical data and compare proportions. p < 0.05 was considered statistically significant.

## Results

### Patient characteristics

Patients’ characteristics are listed in Table 1. Patients’ backgrounds were not significantly different excluding the additional subsequent chemotherapies.

**Table 1:** Patient characteristics.

		FTD/TPI (n = 77), %	REG (n = 57), %	p-value
**Sex**	male	38 (49)	30 (53)	0.841
**Age, year**	Median (range)	68 (40–85)	66 (41–81)	0.378
**ECOG PS**	0/ 1/ 2	53 (69)/ 19 (25)/ 5 (6)	36 (63)/ 18 (32)/ 3 (5)	0.696
**Primary site**	Left	56 (73)	42 (74)	1.000
**Number of metastatic sites**	> 1/ 1	50 (65)/ 27 (35)	41 (72)/ 16 (28)	0.573
**Liver metastasis**	Yes	48 (62)	37 (65)	0.856
***(K)RAS* status**	Wild-type	35 (45)	30 (53)	0.374
**Number of prior regimens**	≤ 2/ 3/ ≥ 4	42 (56)/ 23 (30)/ 12 (16)	25 (44)/ 15 (26)/ 17 (30)	0.053
**Prior systemic chemotherapy**	Pre oxaliplatin	74 (96)	57 (100)	0.359
	Pre-irinotecan	69 (90)	57 (100)	0.021
	Pre-bevacizumab	63 (82)	53 (93)	0.106
	Pre-anti-EGFR	33 (43)	35 (61)	0.034
	Pre-REG	25 (32)	0	
	Pre-FTD/TPI	0	18 (32)	
**Additional subsequent chemotherapy**	Yes	28 (36)	38 (67)	0.003

FTD/TPI, trifluridine/tipiracil; REG, regorafenib; ECOG PS, Eastern Cooperative Oncology Group Performance Status; EGFR, epidermal growth factor receptor.

In the FTD/TPI group, 28 patients received additional subsequent chemotherapy, consisting of 17 and 16 who received REG and other drugs, respectively. In the REG group, 37 patients received additional subsequent chemotherapy; of these, 27 received FTD/TPI, while 14 received other drugs. In the FTD/TPI combination group, 24 patients were PS 0 and only one patient was PS 1, while in the FTD/TPI monotherapy group, 29 patients were PS 0, 18 patients were PS 1, and 5 patients were PS 2, which suggested that better PS may lead to the combination therapy.

### Survival and response to treatment

**Fig 1:**
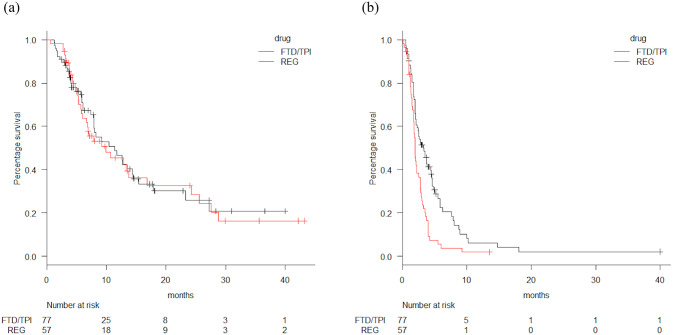
Overall survival (OS) and progression-free survival (PFS) curves of each treatment. (a) OS of treatment groups. Black line = FTD/TPI, trifluridine/tipiracil; red line = REG, regorafenib; median OS = 11.4 vs. 9.9 months; HR, hazard ratio = 0.954 (95% confidence interval, CI 0.606–1.50), p = 0.837. (b) PFS of treatment groups. Black line = FTD/TPI, trifluridine/tipiracil; red line = REG, regorafenib; median PFS = 3.3 vs. 2.0 months; HR, hazard ratio = 0.52 (95% confidence interval, CI 0.357–0.758), p = 0.00055.

**Fig 2:**
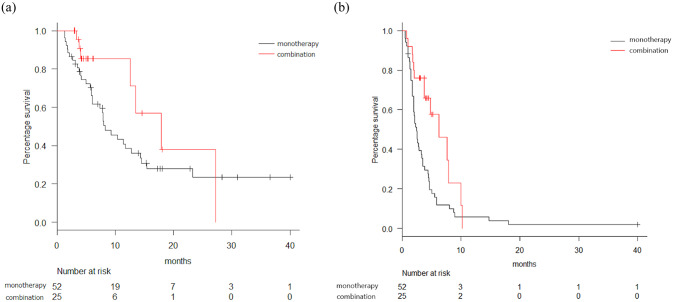
Comparison of overall survival (OS) and progression free survival (PFS) of various treatments. (a) OS curve comparing trifluridine/tipiracil (FTD/TPI) only and combination therapy with molecularly targeted agents. Black line = monotherapy, red line = combination therapy; median OS = 17.9 vs. 8.3 months; HR, hazard ratio = 0.572 (95% confidence interval, CI 0.252–1.30), p = 0.176. (b) PFS curve comparing trifluridine/tipiracil (FTD/TPI) only and combination therapy with molecularly targeted agents. Black line = monotherapy, red line = combination therapy; median PFS = 6.3 vs 2.5 months. HR, hazard ratio = 0.474 (95% confidence interval, CI 0.260–0.864), p = 0.00845.

Tumor responses are shown in Table 2. No patients achieved CR. The ORRs of FTD/TPI and REG were 3% and 2%, respectively (p = 1.000). The DCRs of FTD/TPI and REG were 43% and 32%, respectively (p = 0.406). Survival curves are shown in Fig 1. The median PFS was 3.3 and 2.0 months in the FTD/TPI and REG groups, respectively (hazard ratio [HR] = 0.52, p = 0.00055). The median OS was 11.4 and 9.9 months in the FTD/TPI and REG groups, respectively (HR = 0.954, p = 0.837). As 25 patients in the FTD/TPI group (32%) received combination therapy with molecularly targeted agents (bevacizumab, n = 21; panitumumab, n = 4), we compared the OS and PFS of patients with FTD/TPI monotherapy versus patients using FTD/TPI plus molecularly targeted agents (Fig 2). The median OS was 17.9 and 8.3 months in FTD/TPI combination and monotherapy groups, respectively (HR = 0.572, p = 0.176). The median PFS was 6.3 and 2.5 months in the FTD/TPI combination and monotherapy groups, respectively (HR = 0.474, p = 0.00845). The ORR and DCR were 8% and 72% in the FTD/TPI combination group, respectively while the DCR of the monotherapy group was 29% and no one in the monotherapy group achieved PR. The median PFS was 2.5 and 2.0 months in the FTD/TPI monotherapy group and REG groups, respectively (HR = 0.693, p = 0.069). The median OS between FTD/TPI monotherapy group and REG group revealed no significant difference (8.3 vs 9.9 months, HR = 1.05, p = 0.828). Since our original data include patients who received either REG or FTD/TPI as a prior regimen, we compared those who never received one of these two drugs. The median PFS was 3.8 and 1.8 months in the FTD/TPI group without prior REG treatment (n = 53) and REG group without prior FTD/TPI treatment (n = 40), respectively (HR = 0.428, p = 0.0003). The median OS was 12.7 and 9.9 months in the FTD/TPI group without prior REG treatment and REG group without prior FTD/TPI treatment, respectively (HR = 0.825, p = 0.49).

**Table 2:** Response to treatment.

		FTD/TPI (n = 77), %	REG (n = 57), %	p-value
**Best response**	CR	0 (0)	0 (0)	
	PR	2 (3)	1 (2)	
	SD	31 (40)	17 (30)	
	PD	43 (56)	38 (67)	
	NA	1 (1)	1 (2)	
**ORR**	CR+PR	2 (3)	1 (2)	1.000
**DCR**	CR+PR+SD	33 (43)	18 (32)	0.406

CR, complete response; PR, partial response; SD, stable disease; PD, progressive disease; ORR, objective response rate; DCR, disease control rate.

**Fig 3:**
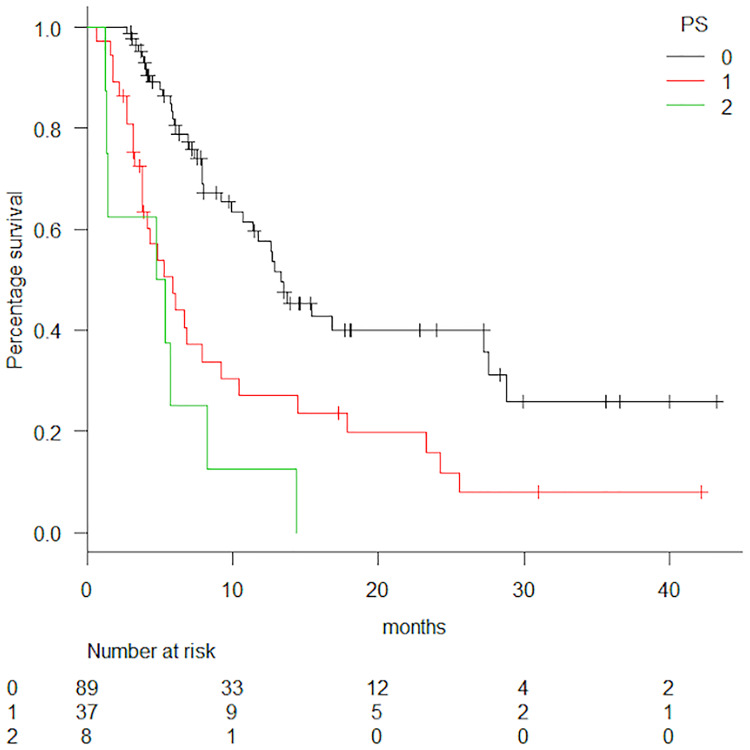
Overall survival (OS) curve classified by performance status (PS). Black line = PS 0, red line = PS 1, green line = PS 2; median OS = 13.4 vs. 5.9 vs 5.1 months. HR, hazard ratio = 2.26 (95% confidence interval, CI 1.626–3.132), p < 0.0001.

**Fig 4:**
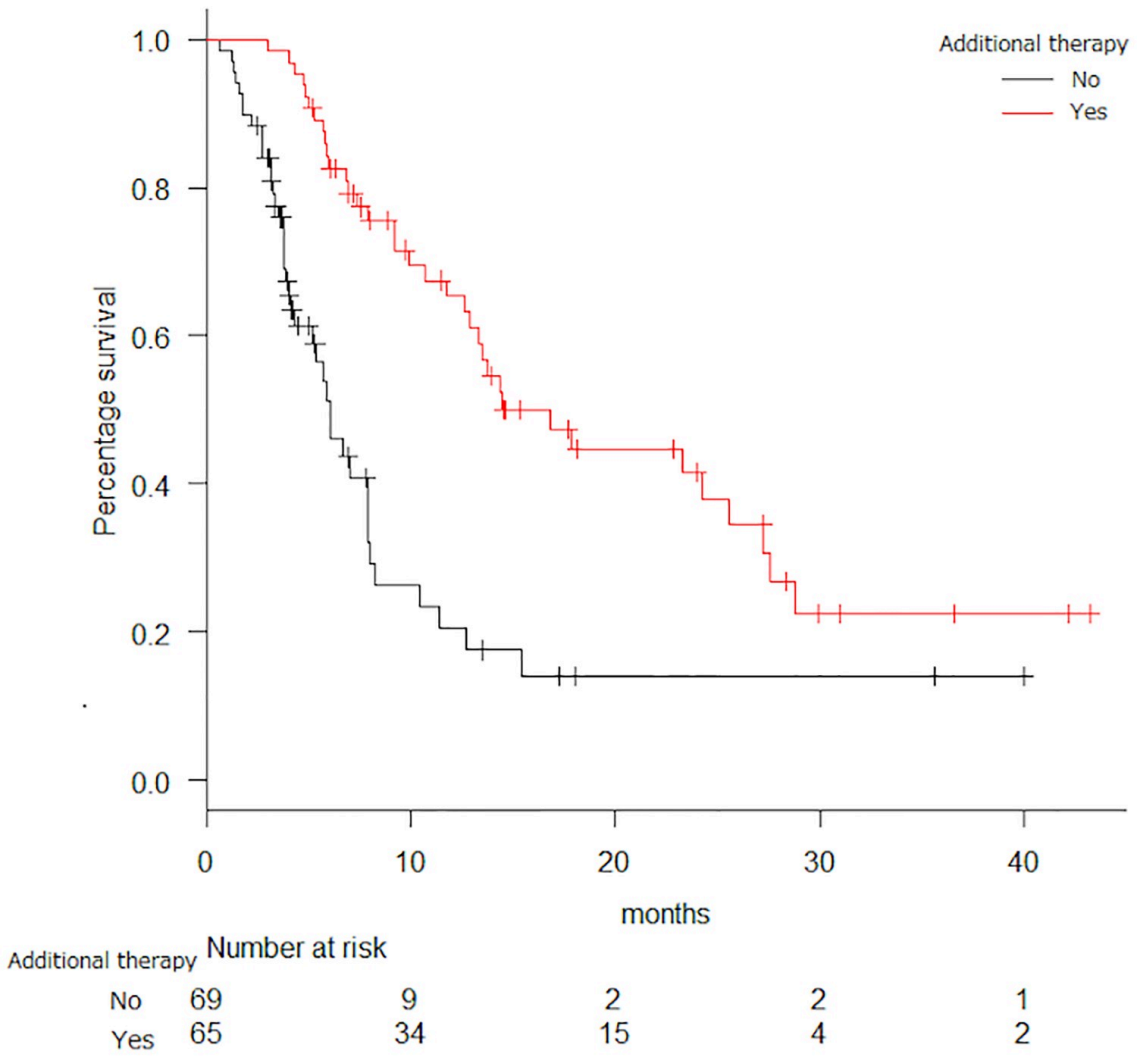
Overall survival (OS) curve for additional therapy. Black line = without additional therapy, red line = with additional therapy; median OS = 16.8 vs. 6.0 months; HR, hazard ratio = 0.34 (95% confidence interval, CI 0.215–0.547), p < 0.0001.

Analysis of the relationship between OS and Eastern Cooperative Oncology Group Performance Status (ECOG PS) revealed that the latter affected the OS, which was 13.4, 5.9, and 5.1 months in the PS 0, PS 1, and PS 2 groups, respectively (HR = 2.26, p < 0.0001). Survival curves classified by PS are shown in Fig 3. Furthermore, the OS of patients administered additional subsequent treatment or not was 16.8 or 6.0 months, respectively, and the difference was statistically significant (p < 0.0001, Fig 4). 30 patients received biological agents as subsequent treatment with median OS of 27.6 months. Of these 30 patients, 21 patients underwent biological agents as re-challenging treatment. 36 patients did not receive biological agents as subsequent treatment with median OS of 12.9 months. Patients with biological agents as subsequent treatment turned out to show significantly better OS than those without biological agents (p = 0.02). The OS of patients with liver metastasis was statistically shorter than that of patients without liver metastasis (p = 0.005). Subgroup analysis of OS revealed no statistical differences by patient age. The median OS for patients aged <65 years and ≥65 years treated with FTD/TPI was 12.6 and 10.4 months, respectively (p = 0.577). The median OS for patients aged <65 and ≥65 years treated with REG was 10.7 and 9.21 months, respectively (p = 0.621). Multivariate Cox regression analysis was performed to examine the effect of clinical features, such as age, ECOG PS, *RAS*, primary site, and additional treatment, on the PFS or OS of each drug. Age and ECOG PS were associated with the PFS of FTD/TPI (p = 0.008, 0.033) and ECOG PS was associated with the PFS of REG (p = 0.049). Additional subsequent treatment was associated with the OS of FTD/TPI (p = 0.004) and additional subsequent treatment and ECOG PS were associated with the OS of REG (p = 0.0003). ECOG PS affected the PFS (p = 0.02), and PS 0 group was the longest. The PFS of patients aged <65 years was shorter than ≥65 years (p = 0.03). The PFS of patients with or without liver metastasis showed no significant difference (p = 0.06). There was no difference in both PFS and OS when classified by gender (p = 0.82, 0.32).

### Adverse events

Table 3 shows the data on drug-related adverse events. The noteworthy FTD/TPI-related adverse event was neutropenia, which occurred in 71% of patients. Grade 3 or 4 neutropenia was reported in 35% patients; however, only 1% experienced febrile neutropenia. The major REG-related adverse events reported were hyperbilirubinemia (42%), hand-foot syndrome, and hypertension.

**Table 3:** Drug-related adverse events.

Grade	FTD/TPI (n = 77)		REG (n = 57)		p-value
	Any, %	≧ 3, %	Any, %	≧ 3, %	
**Hematologic**					
**Neutropenia**	55 (71)	27 (35)	9 (16)	1 (2)	< 0.001
**Anemia**	62 (80)	9 (12)	36 (63)	0 (0)	0.009
**Thrombocytopenia**	30 (38)	4 (5)	29 (51)	7 (12)	0.165
**Febrile neutropenia**	1 (1)	1 (1)	0 (0)	0 (0)	1.000
**Hyperbilirubinemia**	14 (18)	3 (4)	24 (42)	0 (0)	0.001
**Non-hematologic**					
**Anorexia**	34 (44)	3 (4)	29 (51)	2 (4)	0.886
**Diarrhea**	14 (18)	0 (0)	11 (19)	2 (4)	0.520
**Fatigue**	43 (56)	8 (10)	36 (63)	5 (9)	0.783
**Fever**	7 (9)	1 (1)	17 (30)	0 (0)	0.003
**Hand-foot syndrome**	1 (1)	0 (0)	35 (61)	4 (7)	< 0.001
**Hepatic encephalopathy**	0 (0)	0 (0)	3 (5)	3 (5)	0.075
**Hoarseness**	2 (3)	0 (0)	15 (26)	0 (0)	< 0.001
**Hypertension**	13 (17)	2 (3)	36 (63)	8 (14)	< 0.001
**Nausea/vomiting**	25 (32)	0 (0)	5 (9)	0 (0)	0.001
**Proteinuria**	22 (29)	2 (3)	23 (39)	5 (9)	0.217
**Skin disorder**	9 (12)	0 (0)	12 (21)	2 (4)	0.061
**Stomatitis**	15 (19)	0 (0)	4 (7)	1 (2)	0.048

### Dosing patterns

A proportion of REG patients received a reduced-dose treatment from the beginning. The reduced-starting group was statistically younger (p = 0.0189) with a better ECOG PS than the other group. The DCR of the reduced-starting group was 39% (SD response was observed in 7 out of 18 patients of the reduced-starting group), which was similar to that of all patients. The median OS of the reduced-starting group was 16.8 months, whereas that of the standard dosing group was 6.9 months (p = 0.265). The median PFS of the reduced-starting group was 2.5 months, whereas that of the standard dosing group was 1.8 months (p = 0.066). In terms of reduction of the incidence of major adverse events, hyperbilirubinemia was minimized by reducing the starting dose (p = 0.004); however, no statistical differences were observed in hoarseness and hand-foot syndrome, and the incidence of hypertension increased in the reduced-starting group (p = 0.0307).

## Discussion

Our retrospective data analysis demonstrated that FTD/TPI and REG exhibit similar efficacy in patients with mCRC refractory to standard chemotherapy. The median PFS and OS of FTD/TPI monotherapy group and REG group in the present study are in agreement with the PFS of 2.0 months and OS of 7.1 months reported in the phase III RECOURSE trial [5], as well as with the PFS of 1.9 months and OS of 6.4 months reported in the phase III CORRECT trial [3]. However, the DCR in our study analysis was lower than that in previous clinical trials, especially in the REG group [3,5–7] (FTD/TPI; 43% in our study but 44% in the clinical trial, REG; 32% in our study but 41% in the clinical trial). This discrepancy may be attributable to the difference in the timing of the imaging tests. That is, the timing of imaging in clinical practice tends to be delayed compared to that in clinical trials, resulting in disease progression before SD is confirmed.

In this study, the rate of additional subsequent chemotherapy in the REG group was significantly higher than that in the FTD/TPI group: as many as 65% of the patients in the REG group, but only 36% of patients in the FTD/TPI group, received additional subsequent chemotherapy. Our results showed that additional subsequent chemotherapy significantly prolonged the OS and that additional chemotherapy especially with biological agents is more effective for prolonging OS. In summary, these findings indicate that it would be reasonable to propose that the OS of the REG group was as high as that of the FTD/TPI group although the PFS of the REG group was significantly shorter than that of the FTD/TPI group.

Additional sub-analysis revealed that the effect of ECOG PS on PFS and OS was statistically significant; our study concluded that better PS may lead to improved PFS and OS. Comparison between younger and elderly patients revealed no significant differences in OS. A previous multicenter observational study, REGOTAS, reported that subgroup analysis of OS in the REG group tended to be longer in patients aged <65 years (HR, 1.29; 95% confidence interval [CI], 0.98–1.69), whereas that in the FTD/TPI group tended to be longer in patients aged ≥65 years (HR, 0.78; 95% CI, 0.59–1.03) [8]. However, the reason for the shorter PFS of patients aged <65 years is still a question.

As is widely known, combination therapy with cytotoxic and molecularly targeted agents has improved the efficacy of colorectal cancer treatment, and late-line therapy is expected to become a promising combination therapy with molecularly targeted agents, based on the results of a previous phase I/II study of FTD/TPI plus bevacizumab (C-TASK FORCE) [9]. The present study included FTD/TPI plus bevacizumab or panitumumab cases, enabling the comparison of OS and PFS between the FTD/TPI monotherapy group and the FTD/TPI combination group. The OS and PFS of the FTD/TPI combination group tended to be longer than those of the monotherapy group, although no significant difference was observed. The DCR was comparable with that reported in the C-TASK FORCE study.

The safety profiles of FTD/TPI and REG were concordant with those reported in previous studies [3,5]. Major adverse events observed in the FTD/TPI group were bone marrow suppression especially neutropenia and anemia (high-grade accounting for 35 and 12%, respectively). Febrile neutropenia was observed only in 1% of cases and therefore considered to be tolerable. Major remarkable adverse events in the REG group were hypertension, hand-foot syndrome, hyperbilirubinemia, and hoarseness.

Some patients in this study started REG with reduced dose and the reduced-starting group tended to show good prognosis. Patient characteristics in the standard dosing group did not negatively influence the result. Although initiation of treatment with a reduced dose is not officially recommended, regorafenib dose-optimisation can be considered as a reasonable choice according to ReDOS study [10]. The exact reason for the lack of reduction in adverse events is not clear; however, in most participants in the standard dosing group, the dose was reduced in the second or subsequent courses.

This study has some limitations, such as the inclusion of only a relatively small number of patients. Moreover, the study was conducted retrospectively in only a limited number of institutions in Japan. However, no ethnic differences between Japanese and Western patients were reported in either of the phase III trials [3,5].

## Conclusions

In conclusion, the present study demonstrated the efficacy and manageable adverse events of real-world late-line use of FTD/TPI and REG for mCRC treatment in Japan. Our study suggests that REG and FTD/TPI have similar survival effects and sequential usage of both drugs is useful for prolonging OS, although these drugs do not work on tumor shrinkage. It is not confirmed which one should be used first, but as is often mentioned, the PS status is surely the important factor of survival, indicating that drug administration should be conducted based on careful observation of patients’ condition and trying not to worsen their PS. Reduced-dose introduction of REG might be a treatment option to prevent worsening their condition. Future investigations on the optimal sequential order of administration of these two drugs, effectiveness of combination therapy with molecularly targeted agents, and validation of effective starting-dose of REG are anticipated.

## Supporting information

S1 Data (PDF)
