# Supplementary material for: Regorafenib vs trifluridine/tipiracil for metastatic colorectal cancer refractory to standard chemotherapies: A multicenter retrospective comparison study in Japan
Source: PLoS One. 2020 Jun 12;15(6):e0234314. doi: 10.1371/journal.pone.0234314 (PMC7292354; doi:10.1371/journal.pone.0234314)
Supplement: S1 Data — (PDF) [file pone.0234314.s001.pdf]

drug	male=1	age	PS	right=1	No. of metastatic sites
FTD/TPI	1	65	0	0	2
FTD/TPI	0	65	1	0	2
FTD/TPI	1	68	1	0	1
FTD/TPI	1	85	0	1	3
FTD/TPI	0	82	0	0	1
FTD/TPI	1	72	0	0	2
FTD/TPI	1	73	0	0	1
FTD/TPI	0	75	1	0	1
FTD/TPI	1	83	1	1	1
FTD/TPI	1	74	1	1	2
FTD/TPI	0	49	0	0	1
FTD/TPI	1	64	2	0	2
FTD/TPI	1	43	0	0	1
FTD/TPI	0	66	2	1	2
FTD/TPI	1	75	0	0	2
FTD/TPI	1	59	2	0	2
FTD/TPI	1	58	0	0	2
FTD/TPI	0	66	1	0	1
FTD/TPI	0	64	0	0	1
FTD/TPI	0	59	0	0	2
FTD/TPI	1	69	1	0	2
FTD/TPI	0	84	1	1	3
FTD/TPI	1	71	0	0	3
FTD/TPI	0	72	0	0	2
FTD/TPI combination	1	76	0	0	4
FTD/TPI	0	55	1	0	3
FTD/TPI	0	68	0	0	1
FTD/TPI combination	1	77	0	0	4
FTD/TPI combination	0	75	0	0	2
FTD/TPI combination	0	70	0	0	1
FTD/TPI combination	0	68	0	1	2
FTD/TPI	1	68	0	0	2
FTD/TPI	1	66	0	0	3
FTD/TPI	1	57	0	0	2
FTD/TPI	1	68	0	1	2
FTD/TPI	0	78	0	0	3
FTD/TPI combination	0	54	0	1	4
FTD/TPI combination	1	68	0	0	2
FTD/TPI	1	56	0	0	2

FTD/TPI	1	48	0	0	1
FTD/TPI combination	0	64	0	1	4
FTD/TPI combination	0	51	0	0	3
FTD/TPI	0	79	0	1	2
FTD/TPI	1	74	0	1	1
FTD/TPI	1	77	0	0	1
FTD/TPI combination	1	48	0	0	2
FTD/TPI	0	50	0	0	6
FTD/TPI combination	0	56	0	0	1
FTD/TPI	1	68	1	0	2
FTD/TPI	1	65	0	0	1
FTD/TPI combination	0	51	0	0	3
FTD/TPI combination	0	53	0	0	2
FTD/TPI	1	65	0	0	4
FTD/TPI	1	40	0	0	3
FTD/TPI	1	65	1	0	2
FTD/TPI combination	0	59	0	1	2
FTD/TPI combination	0	73	0	1	1
FTD/TPI	1	76	1	0	2
FTD/TPI	1	51	1	1	3
FTD/TPI	0	65	1	0	2
FTD/TPI	0	42	2	0	4
FTD/TPI	0	79	1	1	1
FTD/TPI	1	79	1	0	2
FTD/TPI	0	67	1	0	2
FTD/TPI	1	67	1	1	2
FTD/TPI	0	72	0	1	1
FTD/TPI	0	72	2	1	1
FTD/TPI combination	0	68	1	0	1
FTD/TPI combination	0	66	0	1	2
FTD/TPI combination	1	77	0	0	1
FTD/TPI combination	1	73	0	0	1
FTD/TPI combination	0	71	0	0	1
FTD/TPI combination	0	66	0	0	2
FTD/TPI combination	0	68	0	1	1
FTD/TPI combination	1	67	0	1	1
FTD/TPI combination	0	68	0	0	1
FTD/TPI combination	0	58	0	0	2
REG	1	65	0	0	3
REG	1	59	1	1	3

REG	1	70	1	0	2
REG	1	68	1	0	2
REG	1	72	0	0	2
REG	1	73	0	0	1
REG	1	64	1	0	2
REG	1	70	1	0	2
REG	1	69	1	0	2
REG	0	49	0	0	1
REG	1	43	0	0	1
REG	0	66	2	1	2
REG	1	75	0	0	2
REG	1	59	2	0	2
REG	1	58	0	0	2
REG	1	73	0	0	2
REG	1	65	1	0	2
REG	0	64	0	0	1
REG	0	81	0	0	2
REG	1	71	1	0	1
REG	0	55	0	0	3
REG	0	68	0	0	1
REG	0	70	0	0	1
REG	1	67	0	0	3
REG	1	69	0	1	4
REG	1	58	0	0	2
REG	1	68	0	1	2
REG	0	61	0	0	3
REG	0	64	0	0	1
REG	0	54	0	1	3
REG	1	69	0	0	2
REG	1	56	0	0	2
REG	1	47	0	0	1
REG	0	52	0	0	4
REG	0	79	0	1	1
REG	1	77	0	0	1
REG	1	48	0	0	3
REG	0	50	0	0	6
REG	0	61	0	1	4
REG	0	52	0	0	3
REG	1	65	0	0	4
REG	1	41	0	0	3

REG	0	59	0	1	2
REG	0	66	0	0	3
REG	1	76	1	0	2
REG	0	65	1	0	2
REG	0	75	1	1	2
REG	1	71	1	1	2
REG	1	79	1	0	2
REG	0	66	0	0	2
REG	0	66	1	0	2
REG	0	67	1	1	1
REG	0	72	1	1	1
REG	0	72	2	1	1
REG	0	68	1	0	1
REG	0	72	1	1	1
REG	0	66	0	1	2

	RAS mutation=1	No. of prior regimens	pre oxaliplat
Liv,Lung	0	4	1
Liv,Lung	1	2	1
Lung	0	4	1
Liv,Lung,Peritoneum	0	3	1
Lung	0	2	1
Liv,Lun	0	3	1
Lung	1	3	1
Lung	1	3	1
Peritoneum	1	2	1
Liv,Peritoneum	1	2	1
Lung	1	4	1
Liv,Lun	0	3	1
Lung	0	3	1
Liv,Lung	1	3	1
Liv,Lung	1	2	1
Liv,Bone	0	6	1
Liv,Lung	1	2	1
Liv,Peritoneum,Bone	1	2	1
Liv	0	4	1
Lung,Brain	1	2	1
Liv,Lung	1	7	1
LN,Lung,Liv	0	3	1
LN,Liv,Lung	1	2	1
Liv,Lung	0	2	1
Liv,Lung,LN,Peritoneum	0	3	1
Liv,Lung,LN	0	3	1
Peritoneum	1	2	1
Liv,Lung,Kidney,LN	0	5	1
Liv,Lung	0	3	1
LN	1	3	1
Liv,LN	1	1	1
Lung,peritoneum	0	2	1
LN,Lung,Liv	0	4	1
Liv,Lung	0	1	1
Liv,LN	unknown	5	1
Liv,Peritoneum,ovary	unknown	2	1
Liv,Bone,Ovary,Skin	1	3	1
Lung,LN	0	2	1
Lung,LN	0	3	1

Lung	1	3	1
Lung,LN,Liv,Peritoneum	1	3	1
Lung,Liv,Pleura	1	3	1
Lung,Liv	0	3	1
Liv	0	1	1
Liv	0	2	1
Peritoneum,LN	0	3	1
Liv,Lung,Ovary,LN,Peritoneum,Bone	1	3	1
Lung	1	3	1
Peritoneum、LN	1	4	1
LN	unknown	1	0
Liv,Lung,Peritoneum	0	1	1
Bone,Liv	1	1	1
Lung,Liv,LN,Bone	1	2	1
Lung,Liv,LN	1	1	1
Liv,Lung	1	1	1
LN,Lung	1	2	1
Liv	1	0	0
Liv,LN	0	6	1
Liv,LN,Peritoneum	1	2	1
Liv,LN	0	3	1
Liv,Lung,LN,Peritoneum	1	2	1
Liv	0	2	1
Lung,LN	0	2	1
Liv,LN	1	3	1
Liv,P	1	2	1
LN	1	4	1
Lung	1	2	1
LN	0	2	1
Liv,Lung	1	2	1
Liv	0	2	1
Lung	0	1	0
Lung	1	2	1
Liv,pleura	1	2	1
Lung	1	2	1
Peritoneum	0	1	1
Peritoneum	1	1	1
LN,Colon	0	2	1
Liv,Lung,LN	0	3	1
Liv,Lung,Peri	0	4	1

Liv,Lung	0	4	1
Lung,LN	0	5	1
Liv,Lun	0	2	1
Lun	1	2	1
Liv,Lung	0	3	1
Lung,Brain	1	5	1
Liv,Lung	1	7	1
Lung	1	5	1
Lung	0	4	1
Liv,Lung	1	4	1
Liv,Lung	1	3	1
Liv,Bone	0	7	1
Liv,Lung	1	3	1
Lung,LN	0	3	1
Liv,Lung	0	4	1
Liv	0	3	1
Liv,Lung	unknown	4	1
Liv	1	3	1
Liv,Lung,LN	0	2	1
peri	unknown	2	1
LN	1	2	1
Lung,Liv,LN	0	5	1
Lung,Liv,LN,adrenal	1	1	1
Liv,Lung	0	2	1
Liv,LN	unknown	4	1
Liv,Bone,Peri	0	3	1
Lung	0	3	1
Ovary,bone,Liver	1	2	1
Lung,LN	0	3	1
Lung,LN	0	4	1
Lung	1	2	1
Lung,Pleura,Liv,Bone	1	4	1
Liv	0	2	1
Liv	0	3	1
Peri,LN,Lung	0	4	1
Liv,Peri,LN,Ovary,Bone,Lung	1	2	1
Liv,Lung,Bone,Peri	1	3	1
Liv,Lung,Peri	0	2	1
Lung,Liv,LN,Bone	1	1	1
Liv,LN,Lung	1	2	1

LN,Lung	1	1	1
Ovary,Lung,Peritoneum	0	3	1
Liv,LN	0	5	1
Liv,LN	0	2	1
Liv,LN	1	2	1
Liv,Lung	1	2	1
Lung,LN	0	2	1
Lung,LN	0	2	1
Liv,LN	1	2	1
Liv	0	2	1
LN	1	2	1
Lung	1	3	1
LN	0	3	1
Liv	0	2	1
Liv,Lung	1	1	1

pre irinotecan	pre bevaci	pre EGFR	Pre REG/FTD	additional subsequent chemotherapy	both drugs
1	1	1	1	1	1
1	1	0	0	0	0
1	1	1	1	1	1
1	1	1	0	0	0
1	0	1	0	0	0
1	1	1	1	0	1
1	1	0	1	0	1
1	1	0	0	0	0
1	1	0	0	0	0
1	1	0	0	0	0
1	1	1	1	1	1
1	0	1	0	0	0
1	1	1	0	1	1
1	1	0	1	0	1
1	1	1	0	1	1
1	1	1	1	0	1
1	1	0	0	1	1
1	1	0	0	0	0
1	1	1	1	1	1
1	0	0	0	0	0
1	1	0	1	1	1
1	1	1	0	0	0
1	1	0	0	1	1
0	1	1	0	1	0
1	0	1	0	1	0
1	1	1	1	0	1
0	1	0	1	0	1
1	1	1	0	0	0
1	1	1	0	0	0
1	1	0	1	0	1
1	1	0	0	0	0
1	1	0	0	0	0
1	1	1	0	1	1
1	0	1	0	1	1
1	0	1	1	0	1
1	0	1	0	0	0
1	1	0	1	0	1
1	1	0	0	1	1
1	1	1	0	1	1

1	1	1	1	0	1
0	1	0	0	1	0
1	1	0	0	1	1
1	1	1	1	1	1
0	0	1	0	0	0
1	0	1	0	1	1
1	1	1	0	1	1
1	1	0	1	0	1
1	1	0	0	0	0
1	1	0	0	0	0
0	0	0	0	0	0
1	1	0	0	1	1
1	1	0	0	1	0
1	1	0	1	0	1
1	1	0	0	1	1
1	0	0	0	0	0
1	1	0	1	1	1
0	0	0	0	0	0
1	1	1	1	0	1
1	1	0	0	0	0
1	1	1	1	0	1
1	1	0	0	0	0
1	1	0	0	0	0
1	1	1	1	0	1
1	1	1	1	1	1
1	0	0	0	0	0
1	1	0	1	0	1
1	1	0	0	1	1
1	1	0	0	1	1
1	1	0	1	0	1
1	1	1	0	0	0
1	0	1	0	0	0
1	1	0	0	0	0
1	1	0	0	0	0
1	1	0	0	0	0
0	1	0	0	0	0
0	1	0	0	0	0
1	1	0	0	1	0
1	1	1	0	1	1
1	1	1	0	0	0

1	1	1	0	0	0
1	1	1	1	1	1
1	1	1	0	1	1
1	1	0	0	1	1
1	1	1	0	0	0
1	1	1	0	1	1
1	1	0	1	0	1
1	1	1	0	1	1
1	1	1	1	1	1
1	1	0	0	1	1
1	1	1	1	1	1
1	1	1	0	1	1
1	1	0	1	1	1
1	1	1	0	0	0
1	1	1	0	0	0
1	1	1	0	1	1
1	1	1	1	0	1
1	1	0	1	0	1
1	1	1	0	1	1
1	1	0	0	1	1
1	1	0	0	1	1
1	1	1	1	0	1
1	0	0	0	1	1
1	0	1	1	1	1
1	0	1	0	1	1
1	1	1	0	0	0
1	1	1	0	0	0
1	1	0	0	1	1
1	1	1	1	1	1
1	1	1	1	0	1
1	1	1	0	1	1
1	1	0	1	0	1
1	1	1	0	1	1
1	0	1	1	1	1
1	1	1	1	0	1
1	1	0	0	1	1
1	1	0	0	1	1
1	1	1	1	1	1
1	1	0	0	1	1
1	1	0	1	0	1

1	1	0	0	1	1
1	1	1	0	1	0
1	1	1	0	1	1
1	1	1	0	1	1
1	1	0	0	0	0
1	1	0	0	0	0
1	1	1	0	1	1
1	1	0	0	1	0
1	1	0	0	1	1
1	1	1	0	0	0
1	1	0	0	1	0
1	1	0	1	0	1
1	1	1	1	1	1
1	1	1	0	1	1
1	1	0	0	1	1

OS,month	dead=1	PFS,month	PD=1	best response	Neutropenia	anemia	thrombocytopenia
22.85714	0	5.857143	1	SD	4	1	0
3.75	1	0.75	1	PD	0	1	0
31	0	4.678571	1	SD	2	1	0
7.928571	1	2.75	1	PD	0	1	1
18.03571	0	8	1	SD	1	1	1
7.928571	1	4.357143	1	SD	4	0	0
40	0	40	0	SD	3	1	1
17.25	0	1.5	1	PD	3	1	0
3.857143	0	3.857143	1	PD	2	3	0
3.178571	0	1	1	PD	0	1	1
7.357143	1	2	1	PD	2	0	0
8.285714	1	4.642857	1	PD	2	3	0
27.21429	0	1.714286	1	PD	1	0	0
1.321429	1	0.571429	1	PD	0	1	0
36.57143	0	2.142857	1	PD	2	0	0
1.285714	1	1.285714	1	PD	0	1	0
15.35714	0	2	1	PD	1	1	0
2.5	0	0.5	1	PD	3	2	0
28.32143	0	2.464286	1	PD	2	1	0
7.785714	0	3.321429	1	SD	2	1	0
23.32143	1	18.07143	1	SD	2	3	2
10.39286	1	2.857143	1	PD	3	3	2
5.928571	1	2.107143	1	PD	4	1	0
5.785714	0	1	0	NA	0	0	0
5.214286	0	3.75	1	SD	1	2	2
1.75	1	0.964286	1	PD	0	0	0
11.35714	1	2.607143	1	PD	1	0	0
4.178571	0	3.75	1	SD	2	3	1
4.071429	0	4.071429	0	SD	2	1	0
5.321429	0	4.821429	1	SD	0	0	0
3.821429	1	1.821429	1	PD	0	0	0
2.714286	1	2.571429	1	PD	0	2	4
11.75	1	5.535714	1	SD	3	2	0
17.67857	0	8.928571	1	SD	3	2	0
4.035714	1	2.571429	1	PD	2	2	0
12.71429	1	5.071429	1	SD	4	3	1
3.357143	1	0.75	1	PD	1	2	0
27.25	1	10.25	1	SD	3	2	1
5	1	1	1	PD	0	0	0

6.964286	0	1.5	1	PD	0	0	0
14.60714	0	7.642857	1	SD	4	2	3
13.5	1	6.25	1	PD	3	0	1
9.25	1	3.25	1	PD	4	1	1
6.107143	1	4.571429	1	SD	0	2	0
13.96429	0	1.714286	1	PD	4	2	1
6.285714	0	1	1	PD	0	0	1
7.964286	1	1.75	1	PD	3	1	0
3.071429	0	3.071429	0	SD	1	1	0
6.035714	1	1.714286	1	PD	0	1	0
15.42857	1	14.75	1	SD	4	1	0
18.10714	0	7.857143	1	SD	4	2	1
4.071429	1	2.071429	1	PD	1	2	1
5.678571	1	2.285714	1	PD	3	1	1
7.964286	0	3.5	1	PD	0	1	0
1.821429	1	1.285714	1	PD	0	2	0
6.035714	0	2	1	PD	1	1	0
5	0	5	0	PR	4	1	0
3.142857	1	2.035714	1	PD	2	1	1
5.928571	1	3.535714	1	PD	2	1	0
1.607143	1	1.214286	1	PD	0	0	1
1.428571	1	0.535714	1	PD	0	1	0
4.142857	1	1.5	1	PD	1	1	0
2.178571	1	1.964286	1	PD	0	2	0
14.5	1	5.821429	1	SD	3	2	1
7.857143	1	4.464286	1	SD	0	3	0
3.5	1	2	1	PD	2	2	3
14.35714	1	8.75	1	SD	0	2	0
17.85714	1	10	1	SD	4	3	2
3.964286	0	4	0	SD	2	2	1
2.964286	0	2.964286	0	SD	3	1	4
3	0	3	0	SD	2	1	0
3.964286	0	3.964286	0	SD	3	1	0
4.214286	0	4.214286	0	SD	2	0	0
4.5	0	4.5	0	SD	2	3	1
5.214286	0	5.214286	0	PR	4	1	1
3.714286	0	3.714286	0	SD	4	2	2
12.60714	1	1.714286	1	PD	3	2	1
27.60714	1	4	1	SD	0	0	1
2.75	1	1.25	1	PD	0	0	0

3.607143	0	2.071429	1	PD	0	1	2
40.75	0	3.785714	1	SD	0	1	2
9.892857	1	1.821429	1	SD	1	1	1
43.25	0	2.75	1	PD	0	1	1
6.678571	1	0.071429	1	PD	0	1	0
5.25	1	1.214286	1	PD	0	0	0
3.75	1	1.5	1	PD	0	1	0
13.35714	1	0.75	1	PD	2	0	2
24	0	0.964286	1	PD	0	0	0
4.75	1	2.75	1	SD	1	1	2
33.92857	0	9.25	1	SD	0	1	3
5.75	1	0.25	1	PD	0	1	0
12.85714	0	2	1	PD	0	1	0
3.392857	0	1.071429	0	PR	0	2	3
3.785714	1	2.535714	1	PD	0	2	2
29.89286	0	1.321429	1	PD	0	0	1
2.964286	0	1.714286	1	PD	1	1	1
3.142857	1	1.785714	1	PD	0	1	0
3.035714	1	1.464286	1	PD	0	0	0
12.85714	1	0.5	1	PD	0	0	0
8.821429	0	3.071429	1	SD	0	0	0
5.214286	1	3	1	SD	0	2	0
8	0	6.035714	1	SD	1	2	0
6.964286	0	2.892857	1	SD	1	1	0
5.785714	1	1.607143	1	PD	0	2	0
4.285714	1	1.785714	1	PD	0	0	1
13.53571	0	13.53571	0	SD	0	0	0
6.928571	1	2.25	1	PD	0	2	0
16.78571	1	3.571429	1	SD	0	1	0
3.857143	1	3.285714	1	SD	0	0	0
14.53571	0	5.535714	1	PD	0	0	0
7	1	3.535714	1	PD	0	0	1
13.75	1	4.25	1	SD	1	1	1
11.5	0	2.25	1	PD	0	2	1
4.035714	0	4	1	SD	0	0	0
10.71429	1	2	1	PD	0	0	1
6	1	1.75	1	PD	0	2	0
9.714286	0	2.75	1	SD	1	2	0
7.928571	1	2	1	PD	0	1	1
4.464286	0	2	1	PD	0	1	0

7.535714	0	1.285714	1	PD	0	0	0
7.178571	0	1.428571	1	PD	0	0	2
4.357143	1	0.964286	1	PD	0	0	2
4.821429	1	2.928571	1	PD	0	1	3
0.607143	1	0.607143	0	NA	0	1	4
2.714286	1	1.214286	1	PD	0	1	0
24.25	1	1	1	PD	0	2	0
28.75	1	2	1	PD	0	1	2
25.53571	1	2.142857	1	PD	3	0	3
3.285714	1	0.964286	1	PD	0	0	1
9.214286	1	1.75	1	PD	0	1	3
5.357143	1	1.857143	1	PD	0	2	0
6.857143	1	3.964286	1	SD	0	2	1
5.25	0	4	1	SD	0	2	1
5	0	0.714286	1	PD	0	1	3

febrile neutropenia	hyperbilirubinemia	fatigue	anorexia	skin disorder	hand-foot syndrome
0	0	0	0	0	0
0	0	0	0	0	0
0	0	0	0	0	0
0	0	0	0	0	0
0	0	0	0	0	0
0	0	1	0	0	0
0	0	0	0	1	0
0	0	1	0	0	0
0	0	0	0	0	0
0	0	0	0	0	0
0	0	0	0	0	0
0	0	0	0	0	0
0	0	0	0	0	0
0	0	0	0	0	0
0	0	0	0	0	0
0	1	0	0	0	0
0	0	0	0	0	0
0	0	2	0	0	0
0	0	0	1	0	0
0	0	1	0	0	0
0	0	2	2	0	0
0	0	2	1	0	0
0	0	2	2	0	0
0	0	1	1	0	0
0	0	0	0	0	0
0	0	2	0	0	0
0	0	1	2	0	0
0	0	1	0	0	0
0	0	0	0	0	0
0	0	0	0	0	0
0	3	3	0	0	0
0	1	0	1	0	0
0	0	0	0	0	0
0	0	1	0	1	0
0	2	3	3	0	0
0	0	0	0	0	0
0	0	3	3	0	0
0	1	0	0	1	0
0	0	0	0	0	0

0	0	1	0	0	0
0	0	1	0	0	0
0	1	1	0	0	0
0	0	1	2	0	0
0	1	1	2	0	0
0	0	2	2	0	0
0	0	0	0	0	0
0	0	1	2	0	0
0	0	0	0	0	0
0	0	2	2	0	0
0	0	2	2	0	0
0	0	0	0	1	0
0	0	0	0	0	0
0	0	0	0	0	0
0	0	2	2	0	0
0	0	3	2	0	0
0	0	0	1	0	0
0	1	0	0	0	0
0	0	3	2	0	0
0	0	1	1	0	0
0	3	3	2	0	0
0	3	3	3	0	0
0	0	2	1	0	0
0	0	0	0	0	0
0	0	1	0	0	0
0	0	1	2	0	0
0	1	3	2	0	0
0	1	2	2	0	0
3	0	2	2	1	0
0	0	2	2	0	0
0	0	0	0	1	1
0	1	1	1	2	0
0	1	1	1	0	0
0	0	1	1	0	0
0	0	0	0	1	0
0	0	2	2	0	0
0	0	1	1	1	0
0	0	2	1	0	0
0	1	0	0	0	2
0	1	0	1	0	0

0	2	0	0	0	2
0	2	1	0	2	2
0	1	2	2	0	0
0	0	0	0	2	0
0	1	0	0	0	0
0	0	0	0	1	0
0	0	0	0	0	0
0	0	0	0	0	0
0	1	0	0	0	2
0	2	1	1	0	0
0	1	0	0	1	0
0	0	0	0	0	0
0	0	0	0	0	2
0	1	0	0	1	1
0	2	0	0	2	1
0	0	0	0	0	2
0	0	2	2	0	1
0	0	2	2	0	0
0	0	1	0	0	1
0	0	1	1	0	1
0	0	2	2	1	2
0	2	0	1	0	0
0	0	2	2	0	2
0	0	0	0	0	3
0	0	2	2	0	0
0	1	1	1	0	1
0	1	1	2	0	0
0	0	1	2	0	2
0	0	1	1	0	0
0	0	2	0	0	3
0	0	1	1	0	1
0	1	0	0	0	2
0	1	1	0	0	2
0	0	2	2	0	1
0	0	1	0	0	2
0	0	0	0	0	1
0	0	1	1	0	2
0	0	0	0	0	2
0	0	1	0	0	2
0	0	1	0	0	1

0	0	0	0	0	2
0	1	1	2	2	3
0	1	1	1	0	0
0	0	2	2	2	3
0	0	3	3	0	0
0	2	2	1	3	0
0	0	1	0	0	2
0	2	2	0	0	2
0	1	3	2	3	1
0	1	2	1	0	0
0	0	2	2	0	1
0	1	3	2	0	0
0	0	3	2	1	0
0	1	3	3	0	0
0	0	1	2	0	1

nausea/vomiting	stomatitis	diarrhea	fever	hypertension	hoarsness	hepatic encephalopathy
0	0	0	0	0	0	0
0	0	0	0	0	0	0
0	0	0	0	0	0	0
0	0	0	0	0	0	0
0	0	0	0	0	0	0
0	0	0	0	0	0	0
0	0	0	0	0	0	0
0	0	0	0	0	0	0
1	0	1	0	0	0	0
0	0	0	0	0	0	0
0	0	0	0	0	0	0
0	0	0	0	0	0	0
0	0	0	0	0	0	0
0	0	0	0	0	0	0
1	0	0	0	0	0	0
0	0	0	0	0	0	0
0	0	0	0	0	0	0
0	0	0	0	0	0	0
1	0	0	0	0	0	0
0	0	0	0	0	0	0
2	0	2	0	0	0	0
1	0	0	0	0	0	0
0	0	0	0	0	0	0
0	0	0	0	0	0	0
0	0	0	0	0	0	0
0	0	0	0	3	0	0
1	2	0	0	0	0	0
0	0	0	0	0	0	0
0	0	0	0	2	0	0
0	0	0	0	0	0	0
0	0	0	0	0	0	0
0	0	0	0	0	0	0
0	0	0	1	0	0	0
0	1	2	0	0	0	0
1	1	0	0	0	0	0
0	0	0	0	0	0	0
2	0	0	0	0	0	0
0	1	0	0	1	0	0
0	0	0	0	0	0	0

0	1	0	0	0	0	0
1	0	0	0	0	0	0
0	0	0	0	0	0	0
1	0	0	0	0	0	0
1	1	1	1	0	0	0
1	0	1	0	0	0	0
0	0	0	1	0	0	0
1	1	0	0	2	0	0
1	0	0	0	0	0	0
1	0	1	0	0	0	0
0	0	0	2	0	0	0
0	0	1	0	1	0	0
0	0	0	1	0	0	0
0	0	0	0	0	0	0
2	0	1	0	0	0	0
0	0	0	0	0	0	0
0	0	0	0	0	0	0
0	0	0	0	0	0	0
0	0	0	0	0	0	0
0	0	0	0	0	0	0
0	0	0	0	0	0	0
0	0	0	0	0	0	0
0	2	0	0	0	0	0
0	0	0	0	2	0	0
0	0	1	0	0	0	0
1	0	2	0	0	0	0
2	0	0	0	2	0	0
1	0	0	0	2	0	0
1	2	2	1	2	0	0
2	0	0	0	2	0	0
0	2	0	0	0	0	0
1	0	0	0	3	1	0
0	1	0	3	2	1	0
1	0	0	0	2	0	0
0	1	0	0	0	0	0
2	1	1	0	0	0	0
1	1	1	0	0	0	0
0	1	2	0	0	0	0
0	0	0	1	2	0	0
0	0	0	0	0	0	0

0	0	0	0	2	0	0
0	0	0	0	0	1	0
2	0	0	0	3	0	0
0	1	0	0	2	0	0
0	0	0	0	0	0	3
0	0	0	0	0	0	0
0	0	0	0	0	0	0
0	0	0	1	2	0	0
0	0	0	0	1	1	0
0	0	0	0	0	0	0
0	0	1	0	1	1	0
0	0	0	0	0	0	0
0	0	0	0	0	0	0
0	0	3	0	2	1	0
0	0	0	1	0	0	3
0	0	0	0	1	0	0
0	0	1	0	0	0	0
0	3	2	0	0	1	0
0	0	0	0	2	0	0
0	0	0	0	2	1	0
0	2	0	0	2	1	0
0	0	0	1	2	0	0
0	0	0	1	3	1	0
0	0	0	0	3	0	0
0	0	0	0	2	0	0
0	0	1	1	0	0	0
0	0	2	1	3	0	0
1	0	2	0	2	1	0
0	0	1	1	2	1	0
0	0	0	0	2	1	0
0	0	0	0	1	0	0
0	0	0	0	0	1	0
0	0	0	0	2	2	0
2	0	0	0	3	0	0
0	0	1	0	2	0	0
0	0	0	0	3	0	0
0	0	0	0	2	0	0
0	0	0	1	2	0	0
0	0	0	0	1	0	0
1	0	0	1	1	1	0

0	0	0	1	1	0	0
0	0	0	0	0	2	0
0	0	0	0	0	0	0
0	0	0	0	0	0	0
0	0	0	2	0	0	4
0	0	0	0	0	0	0
0	0	0	0	3	0	0
0	1	0	2	0	0	0
0	0	0	2	0	0	0
0	0	0	0	0	0	0
0	0	0	0	2	0	0
0	0	0	0	2	0	0
2	0	1	2	3	0	0
0	0	3	1	2	0	0
0	0	0	1	2	0	0

proteinuria
0
0
0
0
0
0
0
0
0
0
0
0
0
0
0
0
0
0
0
0
0
2
1
0
2
1
0
0
0
0
0
0
0
0
0
0
2
2
0

0
0
2
1
0
0
1
2
1
0
3
1
0
1
2
1
0
0
0
0
0
0
0
0
0
0
2
0
1
1
1
0
0
3
0
1
0
0
0
0

0
0
0
0
0
0
0
0
1
0
2
0
0
0
0
0
2
0
1
3
1
0
1
2
0
1
3
3
0
0
0
1
2
0
2
2
1
1
1
1

0
0
0
0
0
0
0
0
0
0
3
0
2
0
3
